# Self-organizing scale-free patterns in a phase-modulated periodic connecting system

**DOI:** 10.1186/s13104-019-4149-8

**Published:** 2019-03-05

**Authors:** Tsutomu Matsunaga, Masaaki Muramatsu

**Affiliations:** 1 Research and Development Headquarters, NTT DATA Corporation, 3-3-9, Toyosu, Koto-ku, Tokyo, 135-8671 Japan; 2 Medical Research Institute, Tokyo Medical and Dental University, 1-5-45, Yushima, Bunkyo-ku, Tokyo, 113-8510 Japan

**Keywords:** Self-organizing scale-free pattern, Dynamic equilibrium state, Oscillating fluctuation

## Abstract

**Objective:**

The regularity of scale-free patterns in rank-size relations has been observed in word frequency, city size distribution, firm size distribution, and gene expression. Because of the common emergence of this regularity, understanding its mechanisms has been of great interest. For obtaining the scale-free pattern regularity, various models based on the rich-get-richer mechanism have been proposed; however, the overarching procedure of searching for the “rich” is in disagreement with the locally interacting behaviors seen in the aforementioned natural and social phenomena.

**Results:**

We implemented a computational model of a resource distribution system inspired by observations of word connectivity, which is created by local constraints with periodic and phase modulatory features. Here, we empirically demonstrated that a phase-modulated periodic connecting system can reach a dynamic equilibrium state as the most probable case, with the self-organizing scale-free patterns. The regularity could be a result of the configurational balance in spatiotemporal inequity during the resource distribution process with an adaptive constrained connectivity. Our results suggest that investigations of interferences of oscillating fluctuations in the system will elucidate the autoregulatory dynamic behavior.

**Electronic supplementary material:**

The online version of this article (10.1186/s13104-019-4149-8) contains supplementary material, which is available to authorized users.

## Introduction

The regularity of scale-free patterns [1] has been observed in word frequency [2], city size distribution [3], firm size distribution [4], gene expression [5], and hyperlinks on the World Wide Web [6]. According to this regularity, rank-size relations could be exhibited for which the frequencies of words or the populations of cities follow the same pattern in relation to their rank on a list. The emergence of global regularity is considered the outcome of collective behaviors in an open system that consists of a set of many locally interacting elements [7, 8]. In a system that has a continuous external energy supply, regularity can be observed when the system reaches a stable state, which is a balance between the energy supplied from outside the system and the energy dissipated inside the system [9, 10]. The balance can be implemented by the effects of fluctuations amplified through chain reactions [11] among the interacting elements in a competitive manner [12]. Although the system can spontaneously reach and adaptively self-sustain the stable state [13], understanding the self-organizing behaviors has been of great interest [14, 15].

For regularity, the network model based on the rich-get-richer mechanism [16] was developed in a study of the formation of the World Wide Web network [17] by Barabási and Albert (called the BA model). In the BA model, network node distributions provide scale-free patterns with network growth settings, where a successively increasing node preferentially links to a node having a larger link. Since then, various models based on the rich-get-richer mechanism have been proposed [18, 19]. However, the overarching procedure of searching for the “rich” (called the “hub” in network models) is in disagreement with the behaviors of locally interacting elements observed in the aforementioned natural and social phenomena [20]. To understand the behaviors of a dynamic system, we implemented a computational model for the distribution of resources [21] such as words, people, and money. Then, we explored whether the recursive process of a resource distribution system can reach a stable state with the regularity of scale-free patterns.

## Main text

### Results and discussion

#### Word connectivity in text

We first took words as elements and analyzed the text of the Online Mendelian Inheritance in Man (OMIM) database [22] as an example of a resource distribution system. The OMIM text, which contains 268,006 words and a total word frequency of 15,358,228, was analyzed for connectivity [23] between the words and the subsequent words (see "Methods"). The distributions of words and their connection frequencies are shown in Additional file 1: Figure S1. As expected, the word frequency distribution exhibits a clear linearity on a log-log plot (Additional file 1: Figure S1a), and the scaling exponent (see "Methods") was confirmed to be about one. As the top 20 words (Additional file 1: Table S1a) show, the number of connections is not associated with the word frequency. Interestingly, the connection frequency also exhibits a linear-like relation (Additional file 1: Figure S1b), suggesting that only a few of the combinations can have large connection frequencies. As observed for the top 20 most frequent connections (Additional file 1: Table S1b), word connections are created by local constraints such as compounds, stock phrases, and grammatical rules. These indicate that words are connected by an adaptive regulation with periodic and phase modulatory features.

#### Rank-size relations of resource distribution systems

Considering that the connectivity can give rise to the appearance of the scale-free pattern regularity, we implemented two types of resource distribution systems employing the urn model [24]: a periodic connecting system and a phase-modulated periodic connecting system. In the systems, balls were randomly set in urns arranged in a horizontal number line. In the periodic connecting system, an urn from which a ball is taken and an urn to which the ball is moved, were chosen by a one-dimensional periodical mapping devised by a linear congruential generator [25]. In the phase-modulated periodic connecting system, the urn to which a ball is moved, was adaptively regulated to be an adjacent urn to the right or left of the urn from which the ball is taken, by successively utilizing the one-dimensional periodical mapping (see "Methods" for the procedures of the systems). By using the urn model, a set of urns and the balls in the urns respectively represent the elements and the energy of the elements [26].

**Fig. 1:**
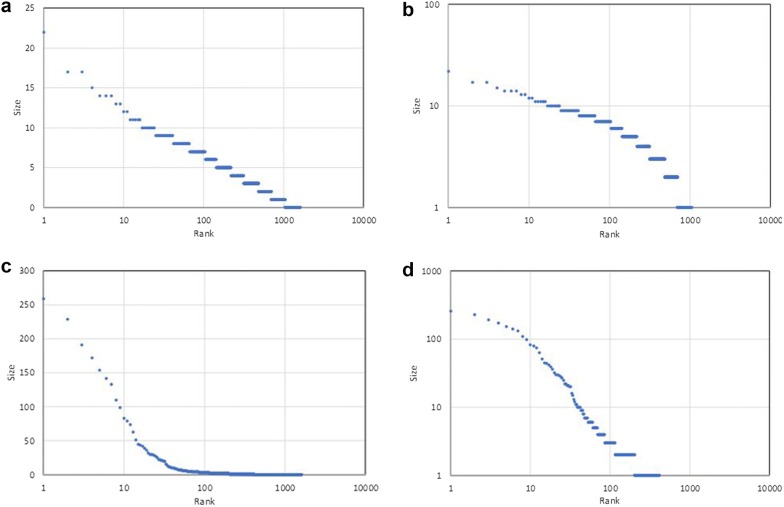
Rank-size relations of number of balls in urns for the case of a total of 3200 balls and 1600 urns (*N* = 3200, *K* = 1600) at the 25-millionth iteration of the **a**,** b** periodic and **c**,** d** phase-modulated periodic connecting systems. In order to comprehend the statistical properties, the rank-ordered frequencies for the systems are plotted in both log-linear coordinates (**a**, **c**) and log–log coordinates (**b**, **d**)

**Fig. 2:**
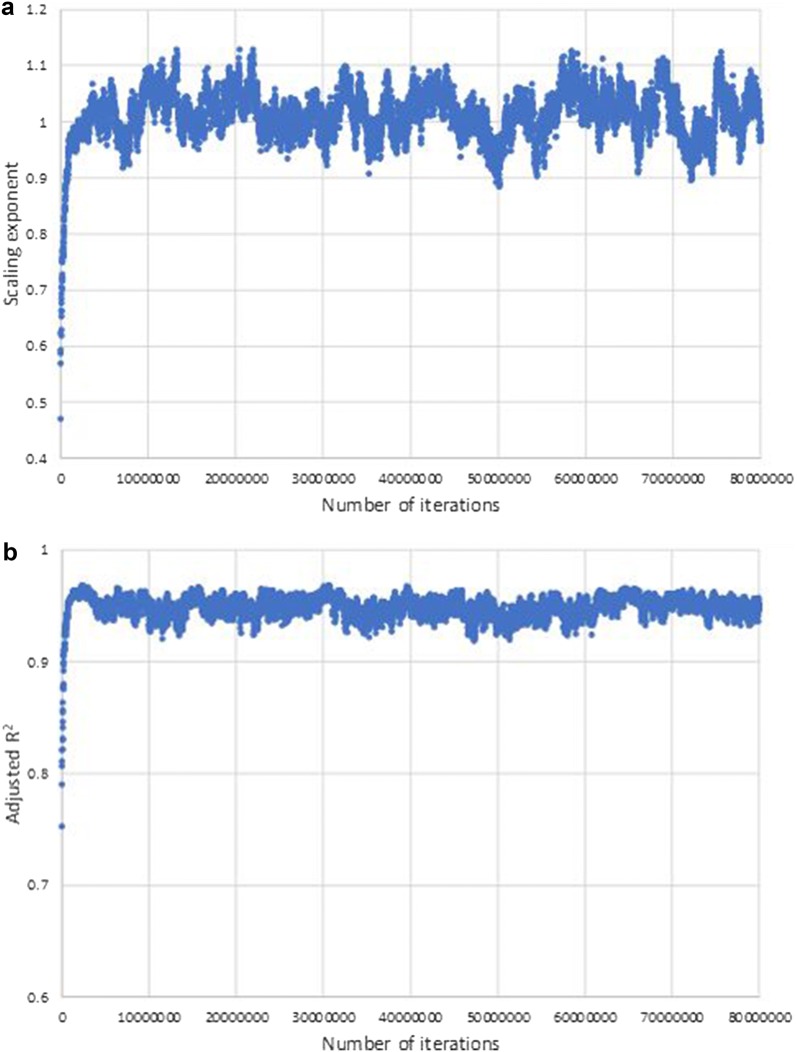
Changes in the **a** scaling exponent and **b** adjusted *R*^2^ of the phase-modulated connecting system (*N* = 3200, *K* = 1600) over 80 million iterations. After about the 5-millionth iteration, the system reaches a stable state through the ball movement process

Figure 1 shows the rank-size relations for the case of a total of 3200 balls and 1600 urns at the 25-millionth iteration. The periodic connecting system corresponds to a closed system of particle elements studied in the field of equilibrium statistical mechanics [10, 26] when the urn choices by the one-dimensional periodical mapping are regarded as being pseudo-random. The appearance of the linearity in the log-linear plot (Fig. 1a) is in agreement with studies where a system has reached and sustained a thermodynamic equilibrium state as the most probable case, having an exponential distribution (known as the Boltzmann distribution) for the energy distribution of oscillating particles through energy exchange among particles [26]. By introducing the phase-modulation, the near linearity in the log-log plot (Fig. 1d) is observed ($\lambda$ (scaling exponent) = 1.15 and $R^2=0.957$), indicating the emergence of the scale-free pattern regularity. The scaling exponent and adjusted $R^2$ (see "Methods") of the phase-modulated periodic connecting system are stable after about the 5-millionth iteration at around one (Fig. 2a) and greater than 0.9 (Fig. 2b), indicating that a dynamic equilibrium state with the formation of scale-free patterns has been self-sustained.

#### Dynamic behavior of the phase-modulated periodic connecting system

To grasp the state transitions of ball distributions for the phase-modulated periodic connecting system, we studied the dynamic behaviors of the numbers of balls in the urns arranged in numerical order. Additional file 1: Figure S2 represents the numbers of balls as bar lengths for the case of a total of 500 balls and 250 urns. From the initial setting in which the numbers of balls in the urns are almost the same (Additional file 1: Figure S2a), the ball movements create such a heterogeneity [27] that balls are admeasured into the modules [28] of the arranged urns (Additional file 1: Figure S2b), and a few urns with large numbers of balls have co-appeared with the larger disparity (Additional file 1: Figure S2c). See Additional file 2: Movie S1 for five million iterations.

**Fig. 3:**
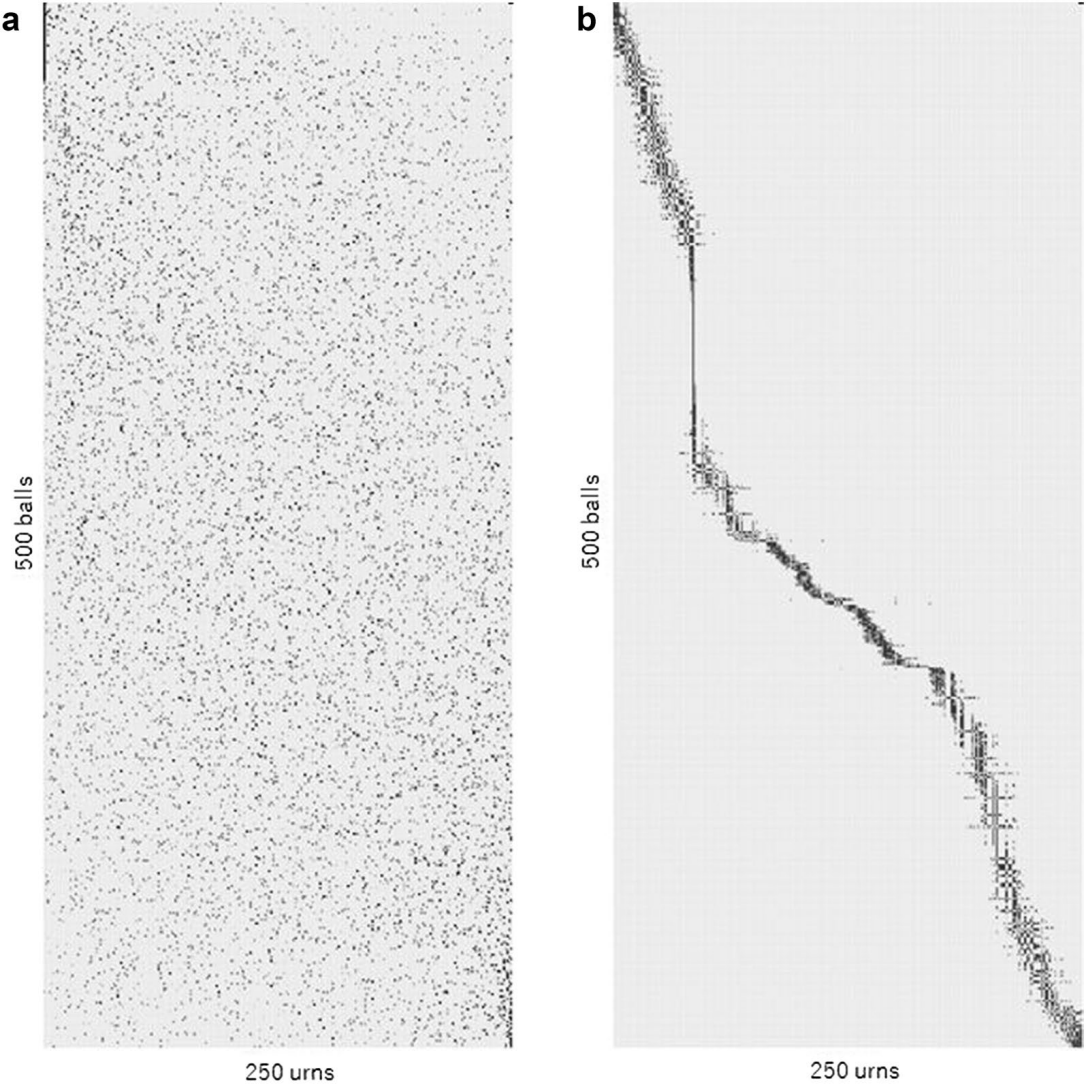
Relations of balls with urns where each ball has been moved (*N* = 500, *K* = 250): the **a** periodic and **b** phase-modulated periodic connecting systems. The plots in grayscale denote the frequencies of the urns where each ball has been moved for 10,000 iterations from the 4.99-millionth to the 5-millionth iteration. The rows (balls) and columns (urns) are sorted in ascending order by the score calculated by correspondence analysis (see "Methods")

Focusing on the movements of each ball, we further investigated the relation of each ball with the urns to which it has been moved. Figure 3 shows the distribution of the urns for each ball during 10,000 iterations from the 4.99- to 5-millionth iteration. In the figure, the plots in grayscale denote the frequencies of the urns to which each ball has been moved, from the top-left to the bottom right with sorting of the rows (balls) and columns (urns) by correspondence analysis [29] (see "Methods"). Whereas the plots of the periodic connecting system (Fig. 3a) are scattered, and the relations of the balls with the urns are independent, the plots of the phase-modulated periodic connecting system (Fig. 3b) show a belt-like relationship, indicating that the balls have successively circulated among their associated urns. This observation is reminiscent of the entrained synchronization of interacting phase-modulated oscillators [30], taking the moving balls as oscillators. We infer that the resonance phenomenon [31] arising in the synchronized ball movement could be relevant to the emergence of scale-free pattern regularity.

The systems we have studied are the so-called complex systems whose behaviors are determined by the current states of the system, and the behaviors define the subsequent states in turn [32]. By employing the urn model, the transient process in a nonlinear dynamic system was studied using the numbers of balls in the urns. Since the development of the BA model, modeling based on the rich-get-richer mechanism that yields the scale-free pattern regularity has been established. Our results indicate a decentralized mechanism [8] by interferences of oscillating fluctuations due to an adaptive constrained connectivity for yielding the self-sustained scale-free pattern regularity. We believe that the dynamic behavior appearing in the phase-modulated periodic connecting system can provide a new perspective on the autoregulation of complex systems; for instance, the autoregulatory popular mobility accompanied by city development.

### Methods

#### Computational models of resource distribution systems

Two types of resource distribution systems, a periodic connecting system and a phase-modulated periodic connecting system, were implemented by employing the urn model [24]. The procedure for the periodic connecting system is as follows:

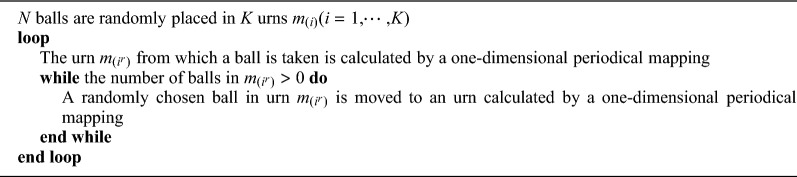



The procedure for the phase-modulated periodic connecting system is as follows:

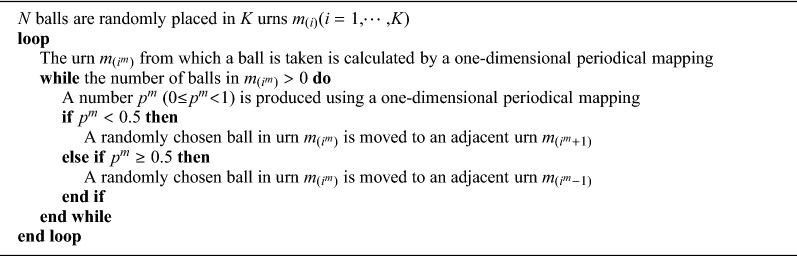



In these systems, a ball can be moved from one urn to another during an iteration. The difference between the two systems is the introduction of a simple adaptive regulation of urn choices in the phase-modulated periodic connecting system. When an adjacent urn has no corresponding urn beyond the boundary, a ball is placed into an urn of another boundary in a circular manner.

A one-dimensional periodical mapping is implemented by adopting a linear congruential generator [25], which is often used to generate pseudo-random numbers. Numbers $P_k$ of period *M* can be generated successively by

$$\begin{aligned} P_{k}=(a{\times }P_{k-1}+c)\,mod M \quad {a,c,M:constant} \end{aligned}$$

The *modM* denotes a remainder operation with *M*. For example, numbers $P_k$ of period $M=16$ are

$$\begin{aligned} 6, 15, 12, 13, 2, 11, 8, 9, 14, 7, 4, 5, 10, 3, 0, 1, 6, 15, 12, \ldots \end{aligned}$$

by setting $a=5,\,c=1, \,P_{0}=1$. As shown above, every number from zero to $M-1$ cyclically appears once in a period. In the experiments, a one-dimensional periodical mapping of period $M=32768$ was used by setting $\hbox {a}=12869$, $\hbox {c}=6925$, $P_{0}=137$.

The urn $m_{(i^{r,m})}$($1\le m_{(i^{r,m})}\le \hbox {K}$) is calculated by the range transformation as follows:

$$\begin{aligned} m_{(i^{r,m})}={K}\times ({P_k}/M)+1 \end{aligned}$$

The number $p^m$($0\le p^m <1$) in the phase-modulated periodic connecting system is provided by dividing $P^m$($0\le P^m < M$) by *M*.

#### Analysis of word connectivity in text

An analysis was performed using the Online Mendelian Inheritance in Man (OMIM) database [22], which is a well-known catalog of human genetic and generic disorders. The text for analysis was taken from a set of “*FIELD* TX” parts of the disease and gene descriptions with the entries numbered from #100050 to #613763 (as of March 2017). The number of words and the total word frequency are 268,006 and 15,358,228, respectively. Here, words are counted including isolated punctuation characters such as commas, full stops, and parentheses. The connections between words were acquired by extracting their adjacent occurrences while ignoring punctuation. Then, the connection frequencies were established by counting the number of connections between the words and the subsequent words. The different connections resulted in 2,192,828 combinations, which correspond to 0.0031% of all possible combinations (268,006 × 268,006 combinations).

#### Analysis of scale-free pattern regularity

For *n* values observed for some phenomenon, $\hbox {X}=\{x_1,x_2,\cdots ,x_n\}$ ($x_1 \ge x_2 \ge \cdots \ge x_n>0$), the relation between *X* and rank *N*

$$\begin{aligned} X={C}N^{-\lambda } \quad {C:constant} \end{aligned}$$

gives the regularity of scale-free patterns, i.e., the so-called power law (called Zipf’s law for word frequency when $\lambda =1$) [1]. Here, $\lambda$ ($\lambda >0$) is a scaling exponent. Scale-free patterns are identified when the rank-size relation forms a straight line on a graph with logarithmic axes. The scaling exponent is estimated by least-squares regression in log–log coordinates. The degree to which the targeted distribution follows the regularity is quantified by using the adjusted $R^2$ ($0\le R^2 \le 1$), which indicates the degree of approximation [4]. A value of $R^2$ greater than 0.7 is considered a good approximation, and it approaches one for a well-approximated distribution.

#### Correspondence analysis

Correspondence analysis [29] is a method used to analyze the relations between variables called cases and items. This analysis yields an arrangement in which similar cases and items are placed close to each other. By introducing a data matrix whose rows and columns are variables (cases and items) with element values depending on the relations between the variables, the rows (items) and columns (cases) are arranged by sorting the scores calculated using the second-largest eigenvalue and the corresponding eigenvector. In the work presented in this paper, items and cases respectively represent balls and urns. The data matrix contains values of the frequencies of the urns to which balls have been moved.

## Limitations

While providing evidence using a decentralized mechanism due to an adaptive constrained connectivity for yielding the scale-free pattern regularity, the current study could not include investigations regarding the consistency of the connectivity with actual dynamics of word occurrences and people movements. Additionally, we do not report on the statistical test [33] for forming power-law distributions, although we have examined scale-free pattern formation using the adjusted $R^2$ in least-squares fitting.

## Additional files

**Additional file 1: Table S1a.** The 20 most frequent words in the OMIM text (terms are sorted in descending order according to their frequencies). **Table S1b.** The 20 most frequent connections in the OMIM text (connections are sorted in descending order according to their frequencies). **Figure S1.** A rank-ordered frequency distribution in the OMIM text: (a) word frequency and (b) connection frequency. The least-squares fitting of the word frequency plot yields *λ* (scaling exponent) = 1.26 and *R*^2^ = 0.993 to obey Zipf’s law. For the connection frequency plot, *λ* (scaling exponent) = 0.909 and *R*^2^ = 0.988 are obtained. **Figure S2.** Examples of distributing a resource of the phase-modulated connecting system for the case of a total of 500 balls and 250 urns (*N* = 500, *K =* 250): (a) initial setting, (b) after 0.5 million iterations, and (c) after 4.8 million iterations. The vertical bars show the number of balls in the 250 urns. The ball movements create such a heterogeneity that balls are admeasured into the three modules which are around 20, 100, and 180 of the numbered urns (b), and then a few urns with larger numbers of balls have co-appeared (c).

**Additional file 2: Movie S1.** Visualization of distributing a resource of the phase-modulated connecting system for the case of a total of 500 balls and 250 urns (N = 500, K = 250). The bar lengths show the number of balls in the urns. The dynamic changes in the numbers of balls are shown up to five million iterations. A few urns having large numbers of balls co-appeared through the ball movement process, whereas the numbers of balls in the other urns were small and fluctuated during iterations.
