# Supplementary material for: Self-organizing scale-free patterns in a phase-modulated periodic connecting system
Source: BMC Res Notes. 2019 Mar 5;12:122. doi: 10.1186/s13104-019-4149-8 (PMC6402156; doi:10.1186/s13104-019-4149-8)
Supplement: Supplementary file 1 — Additional file 1: Table S1a. The 20 most frequent words in the OMIM text (terms are sorted in descending order according to their frequencies). Table S1b. The 20 most frequent connections in the OMIM text (connections are sorted in descending order according to their frequencies). Figure S1. A rank-ordered frequency distribution in the OMIM text: (a) word frequency and (b) connection frequency. The least-squares fitting of the word frequency plot yields λ (scaling exponent) = 1.26 and R2 = 0.993 to obey Zipf’s law. For the connection frequency plot, λ (scaling exponent) = 0.909 and R2 = 0.988 are obtained. Figure S2. Examples of distributing a resource of the phase-modulated connecting system for the case of a total of 500 balls and 250 urns (N = 500, K = 250): (a) initial setting, (b) after 0.5 million iterations, and (c) after 4.8 million iterations. The vertical bars show the number of balls in the 250 urns. The ball movements create such a heterogeneity that balls are admeasured into the three modules which are around 20, 100, and 180 of the numbered urns (b), and then a few urns with larger numbers of balls have co-appeared (c). [file 13104_2019_4149_MOESM1_ESM.pdf]

Self-organizing scale-free patterns in a phase-modulated periodic connecting system

[Additional file 1]

Tsutomu MATSUNAGA,[1*] & Masaaki MURAMATSU [2]

[1]Research and Development Headquarters, NTT DATA Corporation

[2]Medical Research Institute, Tokyo Medical and Dental University

[*]Corresponding author (E-mail: Tsutomu.Matsunaga@nttdata.com)

Mailing address: Toyosu Center Bldg. Annex, 3-9 Toyosu 3-chome, Koto-ku, Tokyo 135-8671, Japan

Table S1a: The 20 most frequent words in the OMIM text (terms are sorted in descending order according to their frequencies).

Rank	Freq.	Word	Incoming	Outgoing
1	763209	.	41595	50016
2	612385	,	55310	54097
3	533358	the	3880	49915
4	495149	of	11002	46105
5	452658	)	50438	6773
6	452564	(	47332	58630
7	420599	and	42549	50398
8	358881	in	20857	20153
9	257766	a	2139	18600
10	201727	et	33974	17
11	201714	al	5	5
12	188781	that	5373	24212
13	187914	to	11595	19101
14	172873	with	8652	19626
15	91885	gene	21382	1918
16	91840	was	13184	4435
17	86351	by	7330	16161
18	84892	is	16276	3466
19	84084	The	468	12234
20	72356	for	5352	11242

The number of incoming and outgoing connections shows the number of words (out of the entire 268,006) of the backward and forward connections, respectively, for each word.

Table S1b: The 20 most frequent connections in the OMIM text (connections are sorted in descending order according to their frequencies).

Rank	Freq.	Connection
1	201700	et → al
2	201641	al → .
3	186617	. → (
4	108558	of → the
5	95551	, → and
6	87767	in → the
7	77183	. → The
8	66467	) → .
9	57168	) → ,
10	39439	that → the
11	29902	. → In
12	27620	to → the
13	26765	, → which
14	25411	) → and
15	20979	) → found
16	20032	, → the
17	19855	of → a
18	19683	in → a
19	19554	found → that
20	19436	. → They

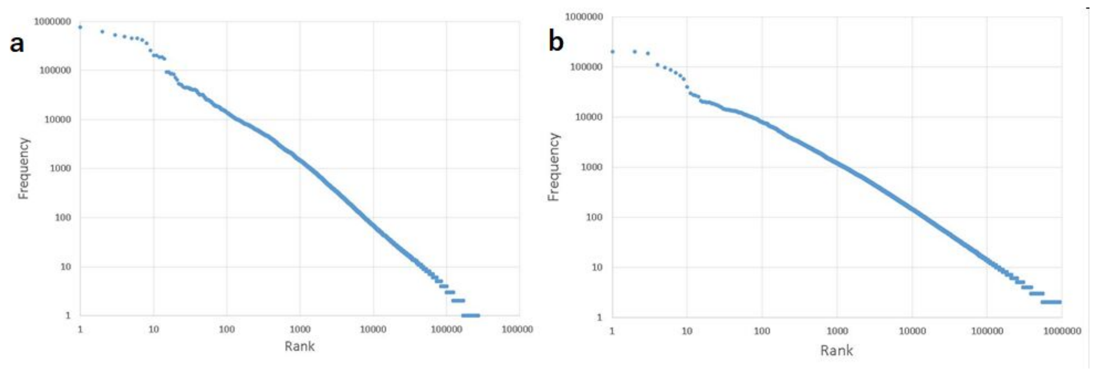

Figure S1: **A rank-ordered frequency distribution in the OMIM text: (a) word frequency and (b) connection frequency.** The least-squares fitting of the word frequency plot yields λ (scaling exponent) = 1.26 and R^2 = 0.993 to obey Zipf's law. For the connection frequency plot, λ (scaling exponent) = 0.909 and R^2 = 0.988 are obtained.

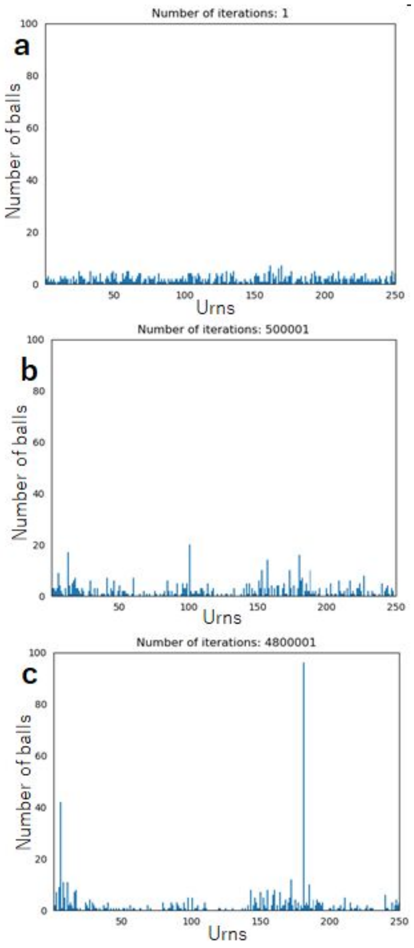

Figure S2: **Examples of distributing a resource of the phase-modulated connecting system for the case of a total of 500 balls and 250 urns (**N **= 500,** K **= 250): (a) initial setting, (b) after 0.5 million iterations, and (c) after 4.8 million iterations.** The vertical bars show the number of balls in the 250 urns. The ball movements create such a heterogeneity that balls are admeasured into the three modules which are around 20, 100, and 180 of the numbered urns (b), and then a few urns with larger numbers of balls have co-appeared (c).
